# Microglia undergo sex-dimorphic transcriptional and metabolic rewiring during aging

**DOI:** 10.1186/s12974-024-03130-7

**Published:** 2024-06-05

**Authors:** Seokjo Kang, Emily Y. Ko, Amelia E. Andrews, Juliana E. Shin, Karina J. Nance, Pijus K. Barman, Peter S. Heeger, Willard M. Freeman, Bérénice A. Benayoun, Helen S. Goodridge

**Affiliations:** 1 Board of Governors Regenerative Medicine Institute, Cedars-Sinai Medical Center, Los Angeles, CA 90048 USA; 2 Research Division of Immunology in the Department of Biomedical Sciences, Cedars-Sinai Medical Center, Los Angeles, CA 90048 USA; 3 Comprehensive Transplant Center, Cedars-Sinai Medical Center, Los Angeles, CA 90048 USA; 4 Genes & Human Disease Program, Oklahoma Medical Research Foundation, Oklahoma City, OK 73104 USA; 5 Oklahoma City Veterans Affairs Medical Center, Oklahoma City, OK 73104 USA; 6 Department of Biochemistry and Molecular Biology, University of Oklahoma Health Sciences Center, Oklahoma City, OK 73104 USA; 7 Leonard Davis School of Gerontology, University of Southern California, Los Angeles, CA 90089 USA; 8 Molecular and Computational Biology Department, Arts and Sciences, USC Dornsife College of Letters, University of Southern California, Los Angeles, CA 90089 USA; 9 Biochemistry and Molecular Medicine Department, USC Keck School of Medicine, University of Southern California, Los Angeles, CA 90089 USA

**Keywords:** Microglia, Aging, Alzheimer’s disease, Sex differences

## Abstract

Microglia, the brain’s resident macrophages, maintain brain homeostasis and respond to injury and infection. During aging they undergo functional changes, but the underlying mechanisms and their contributions to neuroprotection versus neurodegeneration are unclear. Previous studies suggested that microglia are sex dimorphic, so we compared microglial aging in mice of both sexes. RNA-sequencing of hippocampal microglia revealed more aging-associated changes in female microglia than male microglia, and more sex differences in old microglia than young microglia. Pathway analyses and subsequent validation assays revealed a stronger AKT-mTOR-HIF1α-driven shift to glycolysis among old female microglia and indicated that C3a production and detection was elevated in old microglia, especially in females. Recombinant C3a induced AKT-mTOR-HIF1α signaling and increased the glycolytic and phagocytic activity of young microglia. Single cell analyses attributed the aging-associated sex dimorphism to more abundant disease-associated microglia (DAM) in old female mice than old male mice, and evaluation of an Alzheimer’s Disease mouse model revealed that the metabolic and complement changes are also apparent in the context of neurodegenerative disease and are strongest in the neuroprotective DAM2 subset. Collectively, our data implicate autocrine C3a-C3aR signaling in metabolic reprogramming of microglia to neuroprotective DAM during aging, especially in females, and also in Alzheimer’s Disease.

**Supplementary Information:**

The online version contains supplementary material available at 10.1186/s12974-024-03130-7.

## Background

Aging is a major risk factor for neurodegenerative diseases such as Alzheimer’s Disease (AD) and Parkinson’s Disease, and even healthy aging is accompanied by cognitive decline [1]. The underlying mechanisms are poorly understood, but impaired neurogenesis, loss of synapses and increased microglial reactivity are features of the aged brain in both humans and mice [2].

Microglia, the brain’s resident macrophages, are derived from precursors in the yolk sac during primitive hematopoiesis and populate the brain where they are maintained throughout life by self-renewal [3, 4]. Microglia regulate brain development, support neuronal networks, clear debris and apoptotic cells, prune dysfunctional synapses, and repair tissue damage [5]. Altered microglial function is observed during brain aging and thought to contribute to AD development [6]. Neuroprotective phagocytic microglia (disease-associated microglia, DAM) have been reported to arise during brain aging and in AD [7] upon sequential conversion of homeostatic microglia to DAM1 (Trem2-independent) and then DAM2 (Trem2-dependent). However, aging-associated chronic, low-grade inflammation in the brain can result in dysfunctional microglial phagocytosis of cellular debris and lead to amyloid beta deposition in plaques in the AD brain [8], and dysfunctional, proinflammatory subsets of microglia have also been reported during aging [9].

Sex differences are evident in adult microglia [10–12] and in brain aging and AD [13, 14], but it is unclear how sex differences in microglia impact brain aging. Notably, emerging research underscores the potential for microglia to exhibit sex-specific responses when confronted with immune challenges, including infection or injury [10, 15]. These sex-dimorphic responses may underlie varying susceptibilities to neuroinflammatory conditions and, importantly, could contribute to different rates of cognitive decline between males and females. Thus, it is important to understand the mechanisms underlying microglial activation, plasticity and heterogeneity in both sexes throughout life in order to prevent or delay age-related cognitive decline and reduce neurodegenerative disease risk.

In the present study, we first analyzed changes in the transcriptomes of microglia from the hippocampus of young and aged mice of both sexes. Pathway analyses revealed metabolic changes consistent with increased glycolysis in old microglia and implicated elevated AKT-mTOR-HIF1α signaling in these metabolic differences. Notably, the metabolic shift, which we confirmed with Seahorse assays of microglia from the hippocampus and cortex, was more pronounced in females and reflects more abundant DAM in the hippocampus of old females than their male counterparts. Mechanistically, our data indicate that the shift to glycolysis is driven by enhanced production of the complement factor C3a by DAM and autocrine signaling via C3aR. We also observed a similar pattern of elevated glycolysis and complement gene expression in microglia from the 5XFAD model of early onset AD, specifically in the DAM2 subset. Our study sheds mechanistic light on the programming of DAM and highlights the importance of understanding sex differences. Sex-specific approaches to modulate microglial metabolic regulation could pave the way for novel clinical interventions to maintain microglial health and enhance microglial function to preserve cognition during healthy aging and in patients with neurodegenerative diseases.

## Methods

### Mice

Wild-type C57BL/6 mice were from The Jackson Laboratory and the National Institute on Aging (NIA) colony at Charles River Laboratories and were maintained at Cedars-Sinai Medical Center animal facility. For transcriptome analyses, male and female C57BL/6J mice (3- and 24-month animals) were purchased from The Jackson Laboratory. For Seahorse XFp analyses, male and female C57BL/6JNia mice (3- and 22-24-month animals) were obtained from the NIA colony. For histology and western blot analyses, male and female C57BL/6J (3-4-month-old animals) and C57BL/6JNia (23-24-month-old) mice were used. Animals were acclimated in our facility at Cedars-Sinai Medical Center for 2–4 weeks before any processing, and all procedures were performed with Institutional Animal Care and Use Committee approval.

### Microglial isolation from adult mouse brains

Mice were transcardially perfused with ice-cold Ca^2+^- and Mg^2+^-free HBSS (Corning) containing Actinomycin D (5 µg/ml) and Triptolide (10 µM) to prevent microglial activation during ex vivo analysis [16]. Actinomycin D (5 µg/ml), Triptolide (10 µM) and Anisomycin (27.1 µg/ml) were also used during brain dissection, tissue dissociation, myelin removal, and CD11b^+^ microglia isolation steps. After dissecting brains, tissues were dissociated using the Neural Tissue dissociation kit, papain based (Miltenyi Biotec). Following dissociation, cells were filtered through a 70 μm strainer to obtain single cells, and myelin was cleared with Myelin removal beads II (Miltenyi Biotec). Microglia were sorted using Anti-CD11b MicroBeads (Miltenyi Biotec) using AutoMACS. After sorting, cells were pelleted and stored at − 80 °C for further RNA extraction or cells were plated (70 K cells/well) in XFp plates and immediately used for Seahorse XFp analysis.

### Primary microglial culture and C3a stimulation

Primary microglia were obtained from the cerebral cortices and hippocampi of male and female postnatal day 1–2 C57BL6/J mouse pups (bred in house). Meninges were carefully removed in ice-cold Ca^2+^- and Mg^2+^-free Hanks’ balanced salt solution (HBSS; Corning). The tissues were triturated with a glass pipette in Dulbecco’s modified Eagle’s medium (DMEM; HyClone) containing 10% fetal bovine serum (FBS; Sigma-Aldrich) and 1% penicillin, streptomycin, and glutamine (P/S/G; Sigma-Aldrich). Single cells from a brain in 20 ml of culture medium were plated on a poly-D-Lysine (PDL)-coated T-75 flask. After a 24 h incubation at 37℃ in a humidified 5% CO_2_ incubator, culture medium (DMEM with 10% FBS and 1% P/S/G) including 10 nmol/ml mM-CSF was changed to remove floating debris. On day 8 in vitro (DIV8), 4 ml of 10 nmol/ml mM-CSF containing culture medium was added, and on DIV10–11, microglia were isolated from the mixed glial culture by tapping the flask. Isolated microglia were plated on PDL-coated cell culture plates and incubated for 1 day (37℃, 5% CO_2_) before using. Primary microglia were stimulated with vehicle (PBS) or recombinant mouse C3a (10 nM; Peprotech) for the indicated time as in each figure legend. To inhibit glycolysis or the mTOR pathway, primary microglia were pre-treated (1 h) with 5 mM 2-DG (Sigma-Aldrich) or rapamycin (50 nM; Sigma-Aldrich), respectively, prior to C3a (10 nM) treatment for 18 h.

### Measurement of real-time glycoPER and OCR

Real-time glycolytic proton efflux rate (glycoPER) and oxygen consumption rate (OCR) were measured using a Seahorse XFp analyzer (Agilent Technologies) according to the manufacturer’s instructions. Briefly, CD11b^+^ microglial cells isolated from cortex and hippocampus of young (3-month) and old mice (22-24-month) were resuspended in Seahorse XF DMEM medium, pH7.4 (Agilent, #103575-100) or Seahorse XF base medium (Agilent, #103334-100) for Glycolytic Rate assay or Mito Stress assay, respectively. Single cell suspensions were plated on PDL-coated XFp microplates at 70,000 cells per well and used for Glycolytic Rate assay and Mito Stress assay on the same day as cell isolation. For Glycolytic Rate assay of C3a-treated primary young microglia, 45,000 cells were plated on PDL-coated XFp microplates and rested for 1 day (37℃, 5% CO_2_) followed by C3a treatment with or without rapamycin or 2-DG. Basal and compensatory glycolysis were evaluated by treating with rotenone/antimycin A (0.5 µM) and 2-DG (50 mM); basal respiration and ATP production were evaluated by treating with oligomycin (1.5 µM), FCCP (2 µM), and rotenone/antimycin A (0.5 µM) according to the manufacturer’s instructions.

### Western blotting

Lysates from primary microglia or hippocampal brain tissues were prepared in RIPA lysis buffer containing a cocktail of protease and phosphatase inhibitors (Thermo Fisher Scientific, #78,441). Protein concentration was determined by BCA assay kit (Thermo Fisher Scientific). Cell lysates and brain homogenates were resolved on 4–20% SurePage™ gels (GenScript) in MOPS buffer (GenScript) and transferred to a PVDF membrane (Merck Millipore). Membranes were blocked for 1 h at room temperature with 5% skim milk in Tris-buffered saline containing 0.1% Tween-20 (TBST) and incubated overnight at 4 °C with anti-p-AKT (Ser 473, 1:2,000; Cell Signaling Technology, #4060), anti-AKT (1:2,000; Cell Signaling Technology, #4691), anti-p-mTOR (Ser2448, 1:2,000; Cell Signaling Technology, #5336), anti-mTOR (1:2,000; Cell Signaling Technology, #2972), anti-HIF-1α (1:2,000; Novus, #NB100-449), anti-C3aR (1:1,000, Santa Cruz, #sc-133,172), and anti-β-actin (1:1,000; Cell Signaling Technology, #3700) antibodies. After washing, membranes were incubated with the appropriate horseradish peroxidase-conjugated secondary antibody for 1 h at room temperature. Immunoreactive proteins were detected using an enhanced chemiluminescent (ECL) substrate (Thermo Fisher Scientific) and the iBright imaging system (Thermo Fisher Scientific).

### In vitro assessment of fibrillar Aβ_1−42_ phagocytosis

FITC-Aβ_1−42_ peptide (Bachem) was dissolved in 1,1,1,3,3,3-hexafluoro-2-propanol (HFIP; Sigma-Aldrich) to a final concentration of 1 mM. The HFIP was evaporated under a vacuum using Savant SPD2010 SpeedVac Concentrator (Thermo Fisher Scientific), and the dried peptide was stored at -80℃ [17]. Fibrillar Aβ was prepared by dissolving FITC-Aβ_1−42_ peptide to 1 mM in anhydrous dimethyl sulfoxide (anhydrous DMSO; Sigma-Aldrich) followed by dilution to 100 mM in PBS. The resulting solution was then incubated overnight at 37℃.

To assess phagocytosis, primary microglia were incubated with 1 µM FITC-fAβ_1−42_ for 30 min. To remove surface-bound FITC or FITC-Aβ, cells were treated with proteinase K (50 µg/ml; Sigma-Aldrich, #P5568) for 15 min at 4 °C before staining with Zombie Red cell viability assay dye (BioLegend, #423,109). Cells were further incubated with Fc block (2.4G2 cell supernatant) to prevent non-specific antibody binding prior to staining with BUV395 CD11b antibody (BD Biosciences, #563,553). Flow cytometry was performed using an LSRFortessa (BD Biosciences) and data were analyzed with FlowJo 10.9.0.

### Bulk RNA sequencing and pathway analyses

Total RNA was extracted using RNeasy Plus Micro kit (QIAGEN). Library prep and RNA-seq were performed at the Cedars-Sinai Applied Genomics, Computation, and Translational Core. RNA samples were analyzed for their RIN score prior to sequencing. The SMART-Seq V4 Ultra Low RNA Input Kit for Sequencing (Takara Bio USA, Inc., Mountain View, CA) was used for reverse transcription and generation of double stranded cDNA for subsequent library preparation using the Nextera XT Library Preparation kit (Illumina, San Diego, CA). Quantification of cDNA was performed using Qubit (Thermo Fisher Scientific). Indexed libraries were pooled and cleaned up, and the pooled library size was verified on a 2100 Bioanalyzer (Agilent Technologies, Santa Clara, CA) and quantified via Qubit. Sample libraries were sequenced on a NovaSeq 6000 (Illumina) with a 1 × 75 bp read length and coverage of over 30 M reads/sample. Sequence reads were aligned to the mouse reference genome (mm10) using STAR (v.2.7.7a). Differentially expressed genes were identified using the DESeq2 package (v.1.26.0) in R (v.3.6.3; https://www.r-project.org/) with a false discovery rate less than 0.05. Prior to differential expression analysis using DESeq2, we pre-filtered the RNA-seq data to exclude genes with low counts. Only genes with at least six counts across samples were retained. Functional profiling of gene sets was performed using the KEGG database in Gene Set Enrichment Analysis software (https://www.gsea-msigdb.org). To assess canonical pathway enrichment, gene sets were analyzed using the Ingenuity Pathway Analysis tool (QIAGEN IPA).

### Analysis of single-cell RNA-seq data

Single-cell RNA-seq data were downloaded for GEO series GSE98969 [7]. Cells corresponding to CD45^+^ CD11b^+^ Gr1^–^ FACS-sorted samples in 7-wk and 20-mo mice were retained for the aging analysis (Batch descriptions beginning with “young_7w” or “old_20m”), and cells corresponding to CD45^int^ CD11b^int^ Gr1– FACS-sorted samples in 6-mo mice were retained for the Alzheimer’s analysis (Batch descriptions beginning with “AD6m” or “WT6m”). SCTransform, dimensionality reduction, and clustering were conducted using Seurat v4.3.0 [18]. Average expression of gene signatures was calculated using the module scoring function in Seurat. Microglial clusters were identified based on expression of Sall1 and 3 gene sets distinguishing microglia from macrophages [19]. Within microglia, homeostatic microglia and DAM (including the DAM1 and DAM2 subsets) were distinguished based on expression of the gene sets published in the paper that defined them [7], in comparison to Trem2 KO samples from the same study that lack DAM2 cells.

### Immunofluorescence staining and image acquisition

Mice were transcardially perfused with ice-cold PBS, followed by incubation in 4% PFA for 24 h (4℃) and then in 30% sucrose for 72 h (4℃). Cryo-cut coronal brain sections (30 μm thickness) were sequentially immersed in antigen-retrieval solution (Abcam) for 10 min; in mouse-on-mouse blocking reagent (Vector Laboratories) for 1 h; in 5% goat or donkey serum, 0.3% Triton X-100, and 0.5% bovine serum albumin for 10 min; and then incubated overnight at 4℃ with the following primary antibodies: Iba-1 (1:1,000; FUJIFILM Wako Pure Chemical Corporation, #019-19741), Iba-1 (1:1000; NOVUS Biologicals, #NB100-1028), CD68 (1:200; Bio-Rad, #MCA1957), C3aR (1:50; Santa Cruz Biotechnology, #sc-133,172), and p-mTOR (Ser2448, 1:200; Cell Signaling Technology, #5336). Slices were subsequently incubated for 1 h at room temperature with Alexa 488-, 568-, or 594-conjugated IgG secondary antibodies (1:500; Thermo Fisher Scientific), as appropriate, then counterstained with DAPI (0.4 µg/ml) for 10 min. Tissues were imaged using an LSM 780 confocal laser-scanning microscope (Carl Zeiss) with the same laser and intensity settings, and images were exported or analyzed using Zen 3.1 software (Carl Zeiss) and ImageJ software (NIH). Z-stack images were captured (1 μm interval; 11 slices for CD68 and p-mTOR analyses; 13 slices for C3aR analysis) using the ×40 objective lens on a Zeiss LSM780 confocal microscope. Three regions of the hippocampus were analyzed: CA1, CA3 and dentate gyrus. Total numbers of microglia were quantified by counting Iba-1^+^ cells in the hippocampus. CD68 puncta were also evaluated by assessing the proportion of Iba1^+^ cells with intense CD68 staining. C3aR and p-mTOR were evaluated using the ‘RG2B_Colocalization’ plugin in ImageJ (NIH).

### Statistics

Statistical analyses were performed using GraphPad prism 10.0 (GraphPad Software). To assess the normality of the distribution of the raw data, we performed the Shapiro-Wilk test. Comparisons between two groups were performed using two-tailed unpaired *t* tests. One-way ANOVA with Tukey’s post hoc tests was used to compare three or more independent groups. For comparison of multiple factors (e.g. sex and age effect), two-way ANOVA followed by Sidak’s multiple comparison test was used. Data are presented as mean (SD) or mean (SEM) as indicated in the figure legends. *P*-values less than 0.05 were considered statistically significant. Sample sizes were determined based on pilot experiments.

## Results

### Transcriptomic changes during microglial aging are sex dimorphic

**Fig. 1:**
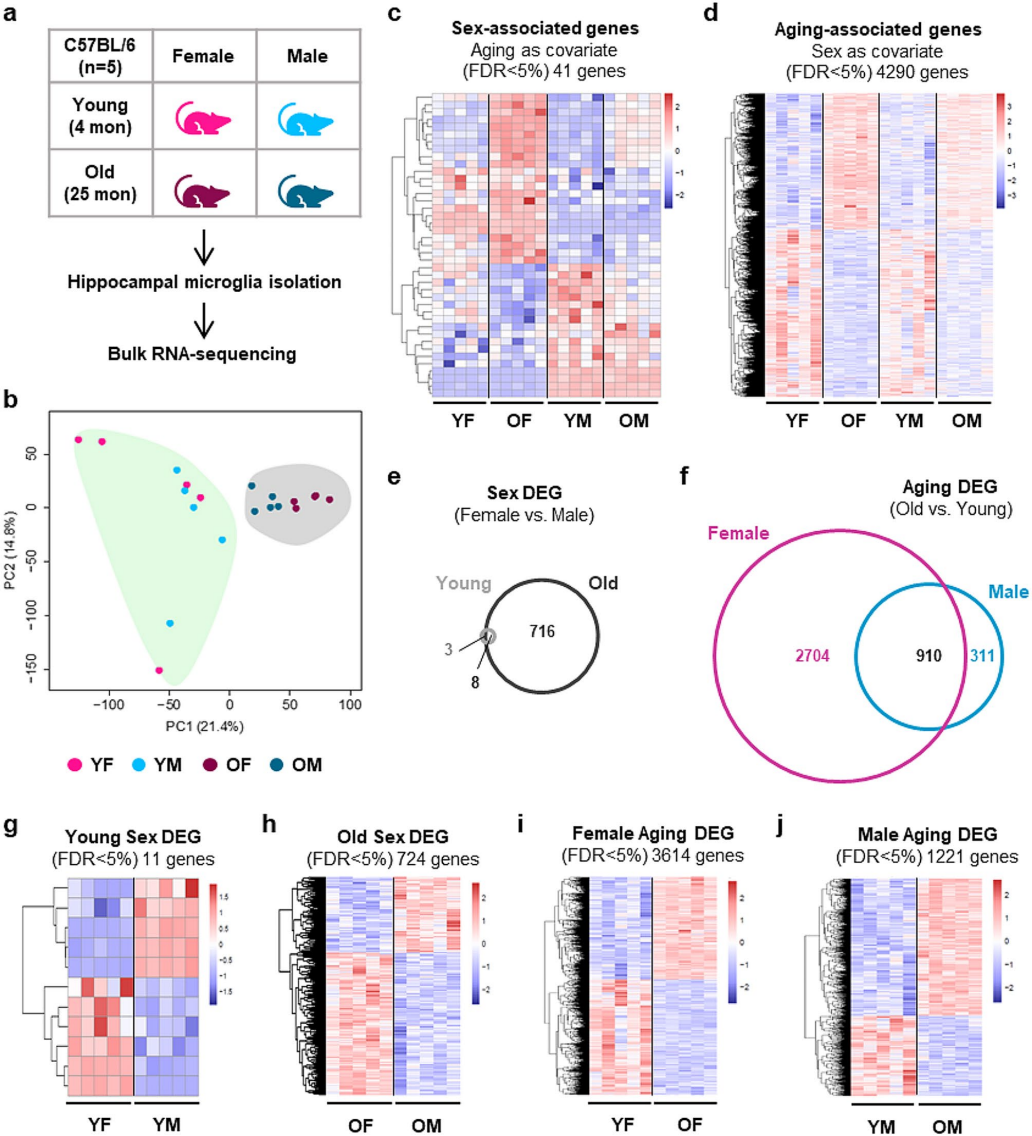
Aging and sex differences in the transcriptomes of hippocampal microglia. (**a**) Scheme of transcriptome analysis of hippocampal microglia from young (4-month) and old (25-month) mice. (**b**) Principal Component Analysis (PCA) of RNA expression by young and old microglia (bulk RNAseq). (**c**) Heatmap of significant (DESeq2, FDR < 5%) sex-associated genes (with age as a covariate). (**d**) Heatmap of significant (DESeq2, FDR < 5%) aging-associated genes (with sex as a covariate). (**e-f**) Venn diagram (plotted using BioVenn [54] of differentially expressed genes (DEG; DESeq2, FDR < 5%) showing the number of Sex DEG in old and young microglia (**e**) and the number of Aging DEG in female and male microglia (**f**). (**g, h**) Heatmaps of Sex DEG (FDR < 5%) comparing young female with young male (**g**) and old female with old male (**h**). (**i, j**) Heatmaps of Aging DEG (FDR < 5%) comparing young female with old female (**i**) and young male with old male (**j**). (Y, young; O, old; F, female; M, male)

To understand how aging impacts microglial gene expression, we performed bulk RNA-sequencing of hippocampal microglia isolated from healthy young (4-month) and old (25-month) mice of both sexes, which allowed us to define differences between young and old, as well as male and female mice (Fig. 1a and Suppl. Figure 1a). Principal component and multi-dimensional scaling analyses revealed clear separation of young and old microglia, with further separation of males and females among the old mice, demonstrating that aging affects the transcriptome of microglia in both sexes, with a stronger effect in females than males (Fig. 1b and Suppl. Figure 1b). Consistent with this, analysis using multivariate linear modeling revealed that age drives stronger variation in gene expression than sex, with 41 sex-associated genes with age as a covariate (FDR < 5%; 23 upregulated and 18 downregulated in female microglia) and 4,290 aging-associated genes with sex as a covariate (FDR < 5%; 1,925 upregulated and 2,365 downregulated in old microglia) (Fig. 1c, d and Additional File 1a, b). Moreover, pairwise analyses revealed more differentially expressed genes (DEG) between old and young in female microglia than in male microglia (Aging DEG; old versus young for each sex), and more Sex DEG (female versus male for each age) in old microglia than young microglia (Fig. 1e-j, Suppl. Figure 1c and Additional File 1c-h).

### mTOR-related pathways are more active in hippocampal microglia during aging, especially in females

**Fig. 2:**
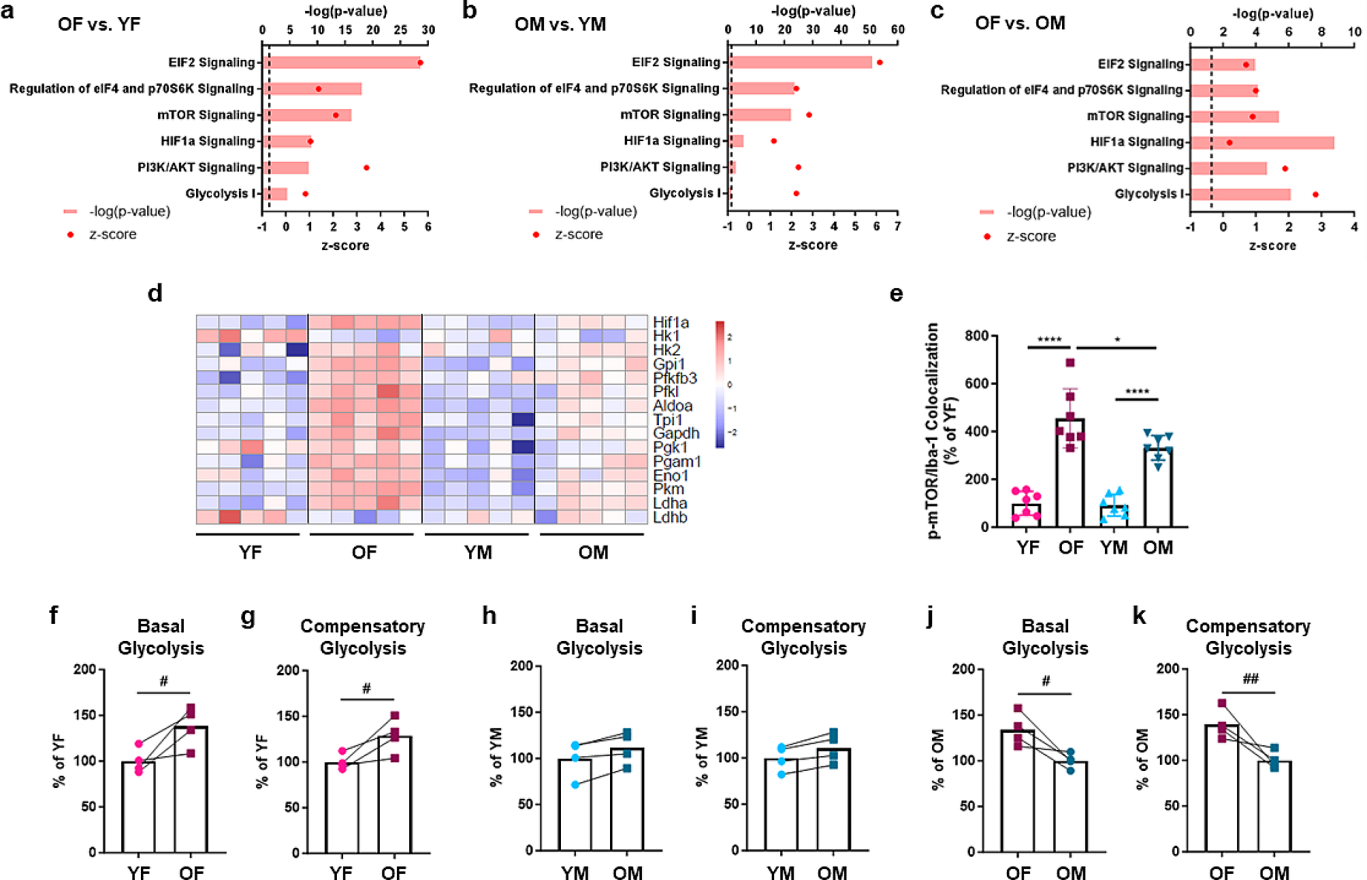
Increased mTOR-related activity and a shift to glycolysis in old hippocampal microglia, especially in females. (**a-c**) Metabolic pathways revealed by Ingenuity Pathway Analysis (IPA) of Aging DEG identified by comparing old versus young female (**a**) and old versus young male (**b**), and Sex DEG in old female versus old male microglia (**c**). Dashed line: -log_10_ (p-value) cutoff of 1.3 (*p* < 0.05). (**d**) Heatmap of *Hif1a* and glycolysis-related gene expression by young and old, female and male microglia (*n* = 5/group). (**e**) mTOR phosphorylation in microglia (Iba-1^+^) in the hippocampus. Quantification of p-mTOR/Iba-1 colocalization (% of YF) is shown. *n* = 7/group. Data are presented as mean (SD). *p < 0.05, ****p < 0.0001 (two-way ANOVA). (**f**-**k**) Microglia from the hippocampus and cortex were pooled and their metabolic activity was assessed using Seahorse assays. Basal and compensatory glycolysis were evaluated by treating with rotenone/antimycin A and 2-DG, and calculating the glycolytic Proton Efflux Rate (glycoPER). Old female microglia were compared to young female microglia (**f, g**), old male microglia were compared to young male microglia (**h, i**), and old female microglia were compared to old male microglia (**j, k**). #*p* < 0.05, ##*p* < 0.01 (unpaired t-test)

We next performed Ingenuity Pathway Analysis (IPA) to predict the functional impact of the differential gene expression. Interestingly, mTOR-related pathways, including PI3K/AKT signaling, which promotes mTOR activation, as well as downstream signaling pathways (eIF2 signaling, and regulation of eIF4 and p70S6K signaling), were significantly different in microglia from old mice versus young mice (aging-associated genes with sex as covariate; Suppl. Figure 2a, b and Additional File 2a) and in old female and old male mice compared their young counterparts (Aging DEG; Fig. 2a, b and Additional File 2b, c). Positive z-scores indicated that these pathways are more active in old microglia than young microglia. Increased mTOR and eIF2/4 signaling is consistent with a recent report of enhanced mTOR-dependent translation of inflammatory proteins in old microglia, in which only female microglia were assessed [20]. Interestingly, our data indicate that these pathways are more active in old female microglia than old male microglia (Sex DEG in old microglia; Fig. 2c and Additional File 2d).

IPA analysis also predicted increased HIF1α signaling and glycolysis in old microglia, with stronger activity of these pathways in old female microglia than their male counterparts (Fig. 2a-c, Suppl. Figure 2b and Additional File 2a-d). Consistent with this, expression of *Hif1a*, another key mTOR target, increased with aging, notably more in female microglia than male microglia (Fig. 2d and Suppl. Figure 2c). We also observed a stronger aging-associated increase in *Hif1a* expression in females in recently published datasets assessing CD11b^+^ hippocampal microglia (RNAseq) and *Cx3cr1*-expressing hippocampal microglia (NuTRAP analysis of translating RNA) [21] (Suppl. Figure 2d).

HIF1α is implicated in cellular metabolic adaptation by inducing glycolysis [22, 23], and metabolic reprogramming plays a crucial role in microglial effector functions [17, 24]. We therefore assessed HIF1α-dependent glycolysis genes and found significantly higher expression of most of the enzymes that catalyze glucose conversion to pyruvate in old microglia, with stronger increases in females than males (Fig. 2d and Suppl. Figure 2c, e). We also observed increased expression of lactate dehydrogenase A (encoded by *Ldha*), which converts pyruvate to lactate to sustain rapid flux through glycolysis, maintaining nicotinamide adenine dinucleotide (NAD^+^) levels in the cell (Fig. 2d and Suppl. Figure 2c). In contrast, expression of lactate dehydrogenase B (encoded by *Ldhb*), which catalyzes the opposite reaction [25], was lower in old female microglia than young female microglia, consistent with more active glycolysis (Fig. 2d and Suppl. Figure 2c). Taken together, our transcriptomic analyses suggested increased activity of mTOR-related pathways in hippocampal microglia during aging, especially in females.

### Microglial metabolism shifts to glycolysis during aging, especially in females

Microglia, like other immune cells, encounter a variety of environmental conditions, requiring them to have a dynamic range of metabolic adaptation pathways. Metabolic pathways are linked to the effector functions of immune cells [26]. For instance, resting macrophages mainly rely on mitochondrial respiration to generate ATP, but during classical activation (e.g. in response to LPS stimulation), macrophage metabolism shifts from oxidative phosphorylation to aerobic glycolysis to facilitate a rapid response. Disrupted metabolic adaptation leads to microglial dysfunction in AD [17], but our understanding of microglial metabolism during healthy aging remains unclear.

Given that mTOR – HIF1α – Glycolysis-related signaling pathways are significantly enriched in old microglia, we next monitored metabolic dynamics of microglia isolated from the hippocampus and cortex of healthy young (3-4-month) and old (22-24-month) mice. We first confirmed increased mTOR phosphorylation in old microglia, especially in female mice (Fig. 2e and Suppl. Figure 3a). We next performed Seahorse XF assays to assess glycolysis and mitochondrial function by real-time measurement of changes in the glycolytic proton efflux rate (glycoPER) and the oxygen consumption rate (OCR), respectively. In female microglia, we observed increased basal and compensatory glycolysis upon aging (Fig. 2f, g and Suppl. Figure 3b). In male microglia, there was a trend towards increased basal and compensatory glycolysis in old microglia, but it was not significant (Fig. 2h, i and Suppl. Figure 3c). Since many of the glycolysis-related genes were more strongly upregulated in old female microglia (Fig. 2d and Suppl. Figure 2c), we also directly compared old female and old male microglia and observed higher basal and compensatory glycolysis in old female microglia (Fig. 2j, k and Suppl. Figure 3d).

We also monitored mitochondrial function. In female microglia, we observed reduced basal respiration and ATP production (Suppl. Figure 3e, f), and in male microglia, there was a significant reduction in ATP production with aging (Suppl. Figure 3 g, h). In line with these differences, we found that several TCA cycle genes were significantly downregulated in old microglia, particularly in females, including *Idh2*, *Idh3a*, *Ogdh*, and *Ogdhl* (Suppl. Figure 3i-k). Interestingly, the downregulated genes are mostly involved in converting isocitric acid to α-ketoglutaric acid, and α-ketoglutaric acid to succinyl CoA; these steps are responsible for generating the electron carrier NADH, which transports electrons to the electron transport chain [27]. Taken together, these data demonstrate a metabolic shift to glycolysis during microglial aging that is more pronounced in females.

### Neuroprotective DAM are glycolytic and more abundant in female mice during aging

**Fig. 3:**
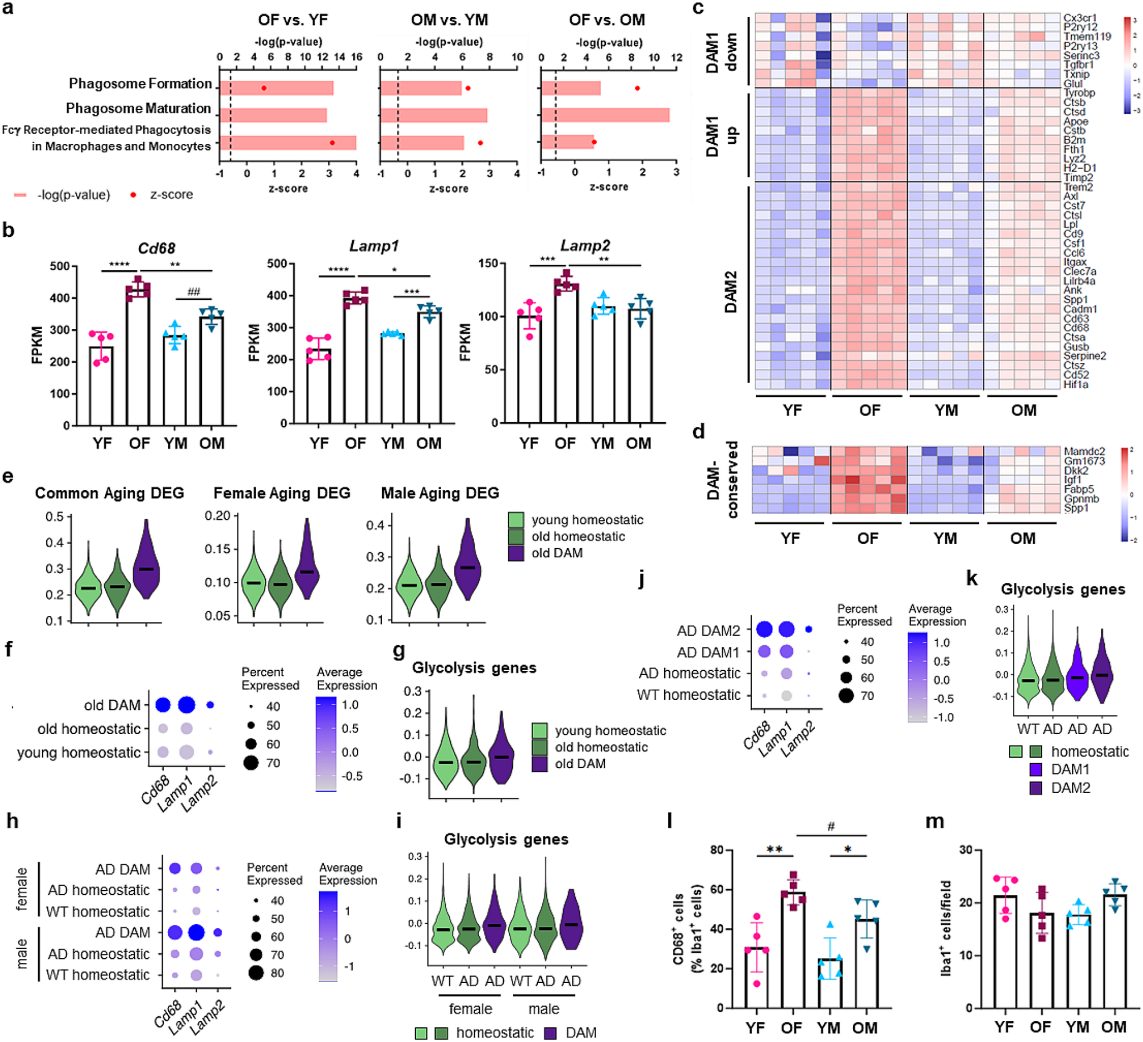
Increased phagocytic activity and more abundant DAM among old female microglia. (**a**) Phagocytosis pathways identified by performing IPA analysis of old versus young female, old versus young male, and old female versus old male microglia. Dashed line: -log_10_ (p-value) cutoff of 1.3 (*p* < 0.05). (**b**) Expression of the lysosomal genes *Cd68*, *Lamp1*, and *Lamp2*. Data are presented as mean (SD) FPKM values. *n* = 5/group. *p < 0.05, **p < 0.01, ***p < 0.001, ****p < 0.0001 (two-way ANOVA). (**c**, **d**) Heatmaps showing expression of DAM1 and DAM2 genes [7] (**c**) and DAM-conserved genes [29] (**d**) by young and old, male and female hippocampal microglia. (**e**) Violin plots with median lines showing expression of Aging DEG common to both sexes, Female Aging DEG, and Male Aging DEG (defined in our bulk RNAseq analysis) by homeostatic microglia (both young and old) and DAM (only prevalent in old mice), assessed by analysis of a previously published scRNAseq dataset (female mice) [7]. (**f**) Lysosomal gene expression by young and old homeostatic microglia and old DAM. Dot size shows percentage of cells expressing the genes, and the color intensity scale indicates average gene expression by all cells in the cluster. (**g**) ‘Reactome_Glycolysis’ gene set gene expression by homeostatic microglia (young and old) and old DAM. (**h**-**k**) Lysosomal gene and ‘Reactome_Glycolysis’ gene set expression by microglia from wild-type (WT) and 5XFAD mice (reanalyzed from [7]). Comparisons of WT and 5XFAD homeostatic microglia with 5XFAD DAM from male and female mice (1 male and 2 females; **h**, **i**), and homeostatic microglia and DAM1 and DAM2 subsets (1 male and 2 females combined; **j**, **k**) are shown. (**l**, **m**) DAM were evaluated by quantifying colocalization of CD68 puncta in hippocampal microglia (Iba-1^+^; **l**); numbers of Iba-1^+^ hippocampal microglia are also shown (**m**). n = 5/group. Data are presented as mean (SD). *p < 0.05, **p < 0.01 (two-way ANOVA); #*p* < 0.05 (unpaired t-test)

IPA analysis also predicted that old microglia of both sexes are more phagocytic than their young counterparts, and that old female microglia are more phagocytic than old male microglia (Fig. 3a and Additional File 2b-d). Consistent with this, the KEGG Lysosome pathway was elevated in GSEA analysis of old female and old male microglia compared to their young counterparts, and in old female versus old male microglia (Suppl. Figure 4a-c and Additional File 2e-g). Notably, we observed stronger upregulation of *Cd68*, *Lamp1* and *Lamp2* expression in females during aging (Fig. 3b).

A subset of neuroprotective microglia known as disease-associated microglia (DAM), which are more phagocytic, increases in neurodegenerative disease models (e.g. AD and ALS), as well as during healthy aging [7, 15, 28]. Both *Cd68* and *Hif1a* are DAM genes [7], so we hypothesized that DAM are more glycolytic and that the stronger upregulation of HIF1α expression and signaling among old female microglia compared to their male counterparts, as well as their higher glycolytic rate, could reflect more abundant DAM in the female hippocampus.

DAM have been reported to form upon sequential conversion of homeostatic microglia to DAM1 and then DAM2 (Trem2-independent and -dependent steps, respectively) [7]. We observed gene expression changes consistent with the formation of DAM1 and DAM2 during aging in our bulk RNAseq dataset, and in recently published transcriptomic and translatomic datasets [21], with stronger differences in females than males (Fig. 3c and Suppl. Figure 4d, e and 5a, b). We also observed stronger upregulation of “DAM-conserved” genes (defined to distinguish neuroprotective DAM from inflammatory microglia) [29] in old female microglia compared to their male counterparts (Fig. 3d).

Assessment of homeostatic microglia (both young and old) and DAM (only prevalent in old mice) in the scRNAseq dataset that defined them [7] (Suppl. Figure 5c, d) revealed that the changes in gene expression we observed in old microglia of both sexes, including the lysosomal genes, were specifically altered in DAM in old mice (Fig. 3e, f). Similarly, glycolysis gene expression was higher in DAM than in homeostatic microglia (Fig. 3g). Evaluation of microglia from the 5XFAD model of early onset AD also revealed higher lysosome and glycolysis gene expression in DAM than homeostatic microglia (Fig. 3h, i and Suppl. Figure 5e, f). These changes were evident in both male and female mice (Fig. 3h, i), and further analysis of the DAM1 and DAM2 subsets revealed that the changes were strongest in DAM2 microglia (Fig. 3j, k).

We therefore performed immunohistochemistry using CD68 as a DAM2 marker [7] to evaluate whether the higher expression of DAM genes in old female microglia reflects a larger proportion of DAM2 in old female mice. We observed more CD68^+^ microglia in old female mice than old male mice (Fig. 3l, m and Suppl. Figure 5 g). Thus, our data collectively attribute the stronger aging-associated metabolic shift among female hippocampal microglia to more abundant DAM.

### C3a – C3aR signaling in microglia promotes glycolysis

**Fig. 4:**
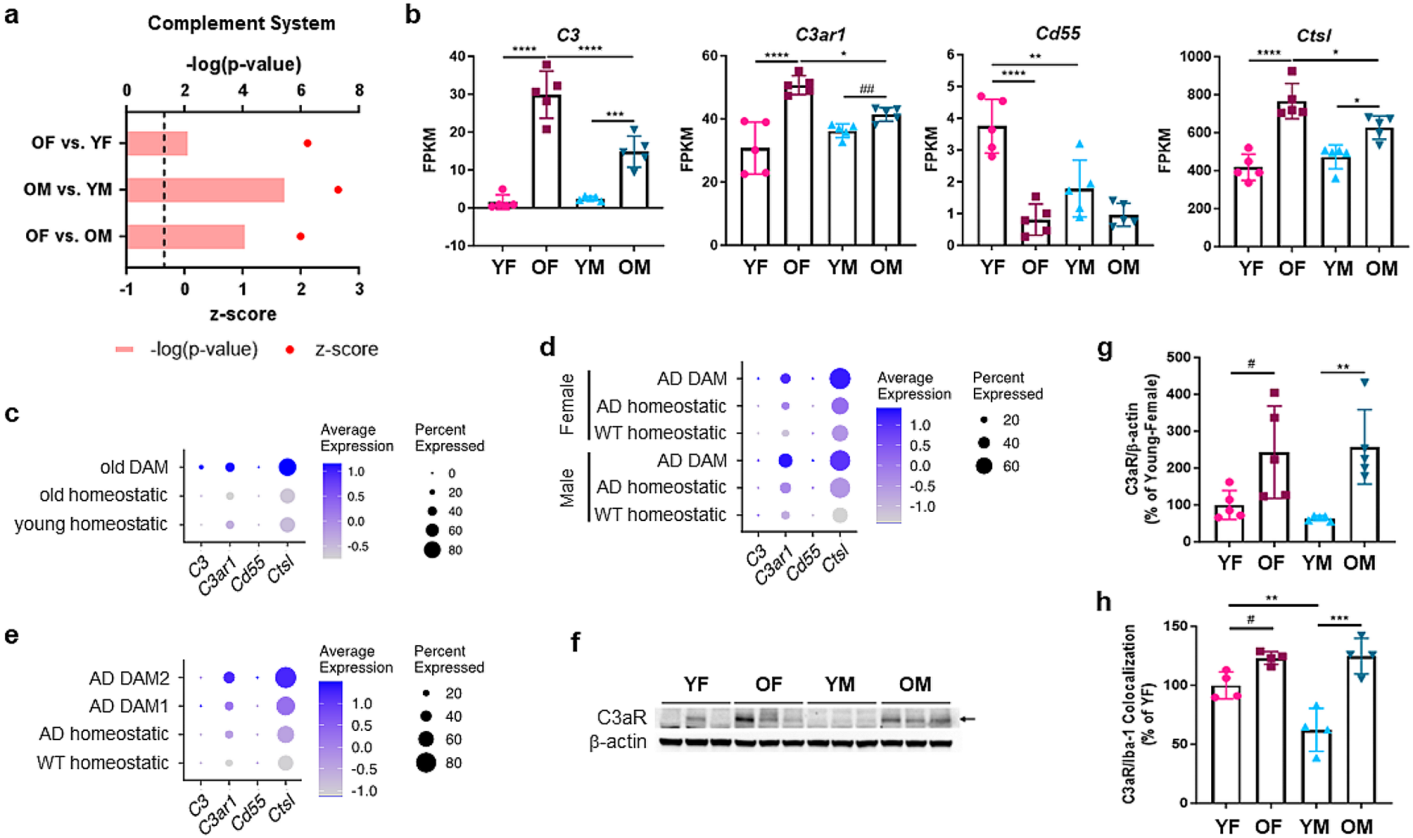
Altered expression of complement pathway components by old hippocampal microglia, especially in females. (**a**) Complement System pathway identified by performing IPA analysis of old versus young female, old versus young male, and old female versus old male microglia. Dashed line: -log_10_ (p-value) cutoff of 1.3 (*p* < 0.05). (**b**) Expression of the complement pathway genes *C3*, *C3ar1*, *Cd55* and *Ctsl*. Data are presented as mean (SD) FPKM values. *n* = 5/group. (**c**) Complement gene expression by homeostatic (young and old) microglia and old DAM [7]. Dot size shows the percentage of cells expressing the genes, and the color intensity scale indicates average gene expression by all cells in the cluster. (**d**, **e**) Complement gene expression by WT and 5XFAD homeostatic microglia and 5XFAD DAM from male and female mice (1 male and 2 females; **d**), and homeostatic microglia and DAM1 and DAM2 subsets (1 male and 2 females combined; **e**) [7]. (**f**, **g**) C3aR protein expression in the hippocampus of young and old, male and female mice. Representative immunoblot images (**f**; arrow: C3aR band) and quantification of C3aR expression (**g**) are shown. *n* = 5/group. Data are presented as mean (SD). (**h**) C3aR expression by microglia (Iba-1^+^) in the hippocampus. Quantification of C3aR/Iba-1 colocalization (% of YF) is shown. *n* = 4/group. Data are presented as mean (SD). *p < 0.05, **p < 0.01, ***p < 0.001, ****p < 0.0001 (two-way ANOVA); #*p* < 0.05, ##*p* < 0.01 (unpaired t-test)

To gain insight into the potential mechanisms underlying altered microglial metabolism during aging, we reviewed our GSEA analysis (Additional File 2e-g) and noticed that ‘KEGG: Complement and coagulation cascades’ is one of the significantly impacted pathways in old microglia (Suppl. Figure 6a-c). IPA analysis also predicted more active complement pathways in old microglia of both sexes, with stronger activity in old female microglia compared to old male microglia (Fig. 4a and Additional File 2b-d). Complement factors, receptors and regulators were all impacted by aging (Fig. 4b and Suppl. Figure 6d, e). *C1qa*, *C1qb* and *C1qc*, which are involved in triggering the classical complement cascade [30], were significantly upregulated in old microglia, particularly in females (Suppl. Figure 6d, e), but most notably, *C3* was dramatically elevated in old microglia of both sexes, and its expression was significantly higher in old female microglia than their male counterparts (Fig. 4b). We also observed similar female-biased aging-associated changes upon analysis of recently published [21] transcriptomic and translatomic datasets (Suppl. Figure 6f, g).

C3 can be cleaved to C3a and C3b by the C3 convertase [31], or by cathepsin L [32]. The gene encoding cathepsin L (*Ctsl*) was increased in old microglia of both sexes and expression of *Cd55*, a C3 convertase inhibitor (also known as decay accelerating factor, DAF) [33], was significantly reduced in old female microglia, with a similar trend in male microglia (Fig. 4b). We also observed increased expression of the C3a and C5a receptors (*C3ar1* and *C5ar1*), as well as the genes encoding CD11c (*Itgax*) and CD18 (*Itgb2*), which combine to form the iC3b receptor CR4 [34] (Fig. 4b and Suppl. Figure 6d, e). The gene encoding the regulatory protein CD59 (*Cd59a*), which inhibits the formation of the membrane attack complex (MAC) on target cells [35], was also significantly downregulated in old microglia, which may suggest an increased probability of MAC formation (Suppl. Figure 6d, e).

Given our observations, we hypothesized that increased *C3* and *Ctsl*, decreased *Cd55*, and increased *C3ar1* would collectively result in increased C3a production and autocrine signaling, so we further evaluated the C3a-C3aR pathway. We observed higher expression of *C3*, *Ctsl* and *C3ar1* in DAM from old mice and 5XFAD mice than their homeostatic counterparts, with notably stronger expression in DAM2 (Fig. 4c-e). Western blotting of hippocampal lysates confirmed significantly increased C3aR protein in both female and male old mice (Fig. 4f, g and Additional File 3a). Microglia are the major cell type expressing C3aR in the mouse brain [36], and immunohistochemistry of the mouse hippocampus verified co-localization of C3aR with Iba-1^+^ microglia and demonstrated significantly increased microglial C3aR expression in old mice of both sexes (Fig. 4h and Suppl. Figure 6 h). Taken together, these data revealed aging-related changes in complement pathway components, consistent with elevated C3a production and C3aR signaling in old microglia, especially in females.

**Fig. 5:**
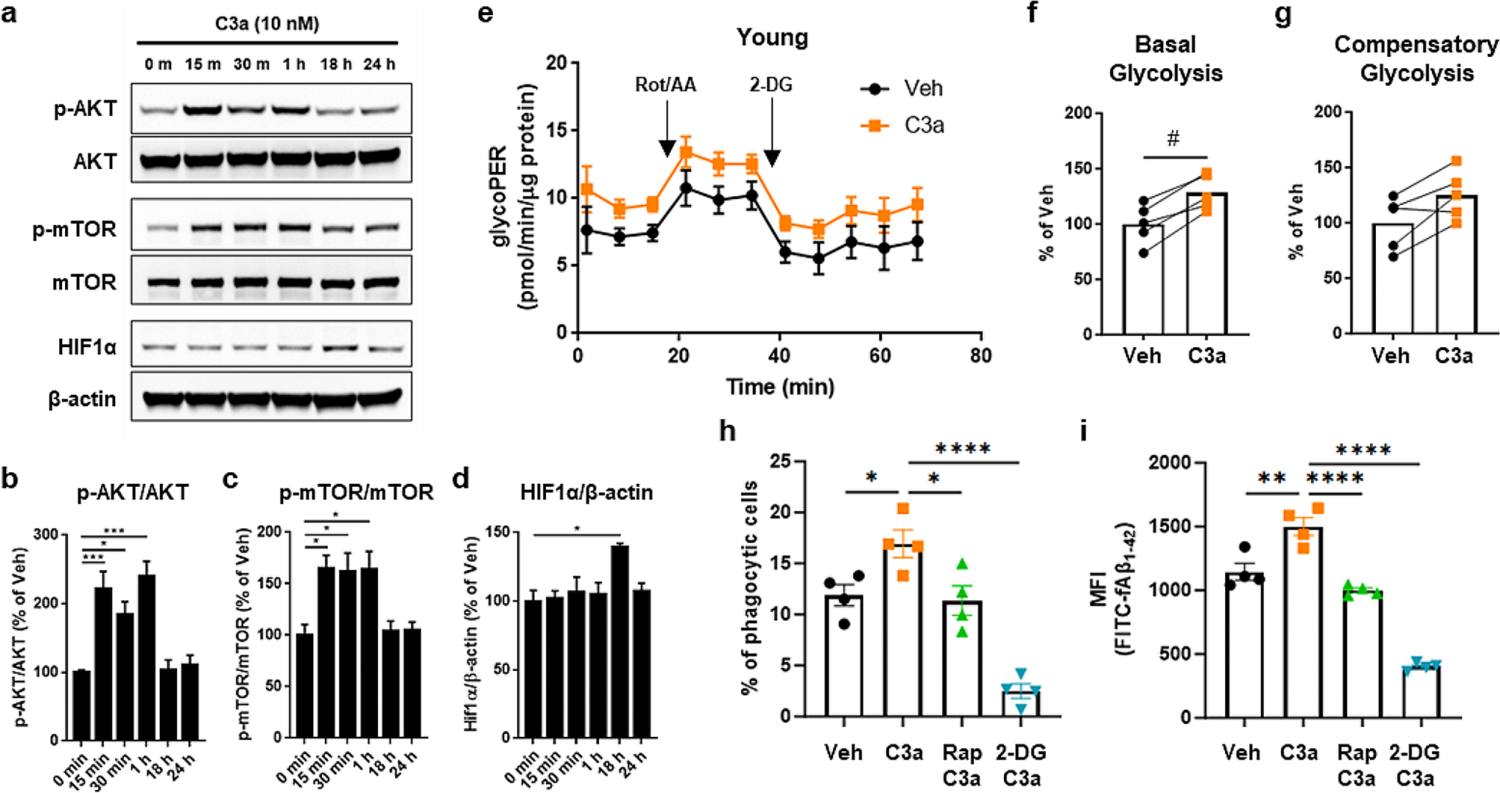
Microglial C3a – C3aR signaling promotes glycolysis and phagocytosis. Young microglia (postnatal day 0–2) were treated with 10 nM recombinant mouse C3a. (**a**-**d**) p-AKT (Ser473), p-mTOR (Ser2448) and HIF1α were assessed by Western blotting at the indicated timepoints (0–24 h), and expression was normalized to total AKT, total mTOR and β-actin, respectively. *n* = 5 replicates. Data are presented as mean (SEM). *p < 0.05, **p < 0.01, ***p < 0.001 (one-way ANOVA). (**e**-**g**) Seahorse assays were used to evaluate real-time glycolytic rate in young microglia (postnatal day 0–2, pooled male and female) after 18 h in vitro treatment with 10 nM recombinant mouse C3a. Stimuli were added as indicated (**e**), and basal glycolysis (**f**) and compensatory glycolysis (**g**) were determined by calculating the glycolytic Proton Efflux Rate (e; glycoPER). *n* = 5/group. #*p* < 0.05 (unpaired t-test). (**h**, **i**) Flow cytometry analysis was performed to assess phagocytosis of FITC-fAβ_1−42_. Rapamycin (50 µM) or 2-DG (5 mM) were pre- (1 h) and co-treated (18 h) with C3a. Proportion of phagocytic cells (**h**) was assessed by evaluating FITC-positive microglia, and FITC MFI was evaluated in total live microglia (**i**). Data are presented as mean (SEM). *p < 0.05, **p < 0.01, ***p < 0.001, ****p < 0.0001 (one-way ANOVA)

In addition to the canonical roles of the complement system, recent studies have suggested that complement proteins can regulate cellular metabolism. For instance, C3a signaling via C3aR activates PI3K/AKT and mTOR in T cells [32, 37] and inhibits oxidative phosphorylation and electron transport chain activity in fibroblasts [38]. To begin to define the impact of C3a on microglial signal transduction and energy metabolism, we treated young (neonatal) microglia with recombinant mouse C3a. Consistent with mTOR activation upon phosphorylation by AKT, C3a treatment increased phosphorylation of both AKT and mTOR at early time points (15 min–1 h), and this was followed by HIF1α induction (Fig. 5a-d and Additional File 3b), which is indicative of mTOR-HIF1α-induced metabolic reprogramming.

We next performed Seahorse XF assays to determine whether C3a-induced mTOR-HIF1α signaling impacts microglial metabolism and observed increased basal glycolysis and a trend towards increased compensatory glycolysis in C3a-treated young microglia (Fig. 5e-g). Collectively, these data implicate elevated microglial C3a – C3aR signaling in the glycolytic shift observed in microglia during aging.

Glycolysis can promote phagocytosis in microglia by providing the necessary energy and metabolites required for target engulfment and digestion [39–41]. Indeed, inhibition of microglial glycolysis can impair their ability to perform phagocytosis effectively [17]. To determine whether C3a-induced glycolysis can also promote the phagocytotic capacity of microglia, we used flow cytometry to evaluate uptake of FITC-fAβ_1−42_ by C3a-treated young microglia. C3a treatment increased the proportion of FITC-fAβ_1−42_-containing microglia, and this effect was abolished by pre- and co-treatment with the mTOR inhibitor rapamycin or the glycolysis inhibitor 2-DG (Fig. 5h, i and Suppl. Figure 7).

Taken together, our studies implicate increased microglial C3a production and autocrine signaling via mTOR-HIF1α-induced glycolysis in the enhanced phagocytic properties of DAM during healthy aging and in an AD model (Suppl. Figure 8). Moreover, our data indicate that this mechanism is activated more strongly in healthy old female mice, which have more abundant DAM than their male counterparts.

## Discussion

Disruption of microglial function has been implicated in the process of brain aging, characterized by the emergence of chronic, low-grade inflammation that is closely associated with neurodegenerative disorders such as Alzheimer’s disease (AD) [6, 8]. Neuroprotective microglia (DAM) have also been proposed to limit pathology by clearing cellular debris and other threats [7], but little is known about sex differences in microglia, including DAM. Our RNAseq analysis, along with a recent report evaluating the transcriptomes and translatomes of hippocampal microglia [21], demonstrated more sex differences in old microglia than young microglia and a stronger impact of aging on female microglia than male microglia.

A recent study implicated enhanced AKT-mTOR-dependent protein synthesis in the inflammatory responses of microglia in old female mice [20], but did not evaluate males. Our data demonstrate stronger aging-associated mTOR activation in females than males, and further reveal that this results in a HIF1α-driven metabolic shift to glycolysis that is specific to DAM. Interestingly, we observed more abundant DAM in old females than old males, which is consistent with a recent report of more Clec7a^+^ CD11c^+^ microglia in old female mice than their male counterparts [21].

DAM have been identified in both healthy aging and disease states, and reported to restrict AD development by phagocytosing Aβ [7] and limiting accumulation of insoluble tau aggregates in the neocortex [28]. Consistent with the sex dimorphism we observed in healthy aging, bulk analysis of microglia from APP/PS1 mice (an early onset AD mouse model) revealed a stronger metabolic shift towards glycolysis in females than males [42], which may also reflect a greater abundance of DAM. Consistent with a neuroprotective role for mTOR in microglia, a recent study reported that increasing microglial mTOR activation by deleting *Tsc1* enhanced Aβ plaque clearance in 5XFAD mice [43]. Interestingly, *Tsc1* deletion induced expression of *Trem2* and other DAM genes (e.g. *Clec7a, Cd68*, *Ctsd*, *Ctsz*, etc.), which suggests that mTOR may be required for DAM induction. Our analysis of DAM in the 5XFAD model of AD indicates that the glycolytic shift is a specific feature of the DAM2 subset. Future studies will be required to determine whether more abundant glycolytic DAM2 are consistently observed in females in neurodegenerative disease models.

Complement proteins have emerged as key regulators of brain health [34, 44] and cellular metabolism [45], and higher concentrations of C3a have been reported in the cerebrospinal fluid of healthy older humans and AD patients [46]. Our data provide novel insights into the involvement of microglial C3a-C3aR in orchestrating metabolic reprogramming during healthy aging, and support a role for C3a in increasing the phagocytic capacity of DAM. Increased C3 cleavage would also provide C3b and iC3b, which could promote phagocytosis by opsonization. Moreover, our data suggest that C3a production and signaling are stronger in female microglia and correlate with emergence of the DAM subset, although further study is required to specifically determine whether microglial C3a-C3aR signaling drives DAM formation.

It is possible that microglial C3aR signaling originates from an intracellular location rather than at the cell surface because recent studies have demonstrated intracellular C3aR signaling from the lysosome and mitochondria [32, 38, 47]. For instance, cathepsin L cleaves C3 stored in lysosomes, and intracellular C3a generation and lysosomal C3aR signaling support resting T cell survival [32]. We observed increased *Ctsl* expression by old microglia, but also decreased expression of *Cd55*, which would be predicted to increase C3 cleavage by the extracellular C3 convertase, so further studies are necessary to determine whether intracellular versus extracellular C3 cleavage and C3aR signaling increase during microglial aging and DAM formation.

C3a has previously been implicated in neurodegenerative disease. Consistent with our findings, a recent study reported C3aR-dependent activation of HIF1α signaling in microglia and described a C3aR-dependent reduction in mitochondrial respiration and elevated lipid species in microglia of APP-KI AD model mice, although this was demonstrated using global deletion of C3aR, which is expressed by vascular endothelial cells in addition to microglia [48], so it is currently unclear whether these effects are direct or indirect. In contrast, inhibition of C3aR reduced glial activity and rescued neuronal defects in a tauopathy model (PS19 mice) [49], and C3aR antagonist-treated APP-transgenic mice had a reduced plaque burden compared to control AD mice [50]. However, these studies also did not specifically target C3a signaling in microglia. Further investigation is therefore necessary to define the roles of C3aR signaling in healthy aging versus neurodegenerative disease, and at early versus late stages of the aging/neurodegenerative process. Mechanisms that are initially beneficial may become detrimental over time or in the context of more severe damage.

It is currently unclear whether the sex dimorphism we observed is governed by cell-intrinsic or extrinsic mechanisms mediated by sex chromosomes (genetic/epigenetic) or sex hormones, but estrogen and testosterone have been shown to influence microglial activity, synaptic density, and cognitive performance [51]. While both males and females experience hormonal changes as they age, the extent and nature of these changes are more pronounced in females, particularly during menopause [52, 53]. Understanding the interplay between sex hormones, microglia, and synaptic plasticity will provide insights into sex-specific cognitive changes during aging.

## Conclusion

In conclusion, our study has shed light on how aging and sex impact microglia in the context of brain health and revealed mechanisms underlying microglial changes during aging. Our findings not only advance our understanding of microglial dynamics during aging, but also uncover potential therapeutic avenues to prevent or reverse neurodegeneration. By targeting the autocrine C3a-C3aR signaling pathway in microglia, interventions could be developed to mitigate aging-related neurodegenerative processes, promote neuroprotection, and preserve brain function. However, future studies are required to determine how C3a signaling in microglia impacts neuronal health and pathology, and whether such interventions are likely to be equally effective in males and females, given the sex differences we observed.

### Electronic supplementary material

Below is the link to the electronic supplementary material.

Supplementary Material 1

Supplementary Material 2

Supplementary Material 3

Supplementary Material 4

## Data Availability

Datasets supporting the conclusions of this article are available in the GEO repository (https://www.ncbi.nlm.nih.gov/geo/): bulk RNAseq dataset generated for this study (GSE267529), RNAseq and NuTRAP data (GSE233400) and scRNAseq data for homeostatic microglia and DAM (GSE98969). Other datasets supporting the conclusions of this article are included within the article and its additional files.

## Abbreviations

AD: Alzheimer’s Disease
DAM: Disease-associated microglia
DEG: Differentially expressed genes
GlycoPER: Glycolytic proton efflux rate
IPA: Ingenuity Pathway Analysis
OCR: Oxygen consumption rate
