# Supplementary material for: Microglia undergo sex-dimorphic transcriptional and metabolic rewiring during aging
Source: J Neuroinflammation. 2024 Jun 5;21:150. doi: 10.1186/s12974-024-03130-7 (PMC11155174; doi:10.1186/s12974-024-03130-7)
Supplement: Supplementary file 1 — Supplementary Material 1 [file 12974_2024_3130_MOESM1_ESM.pdf]

Additional File 3

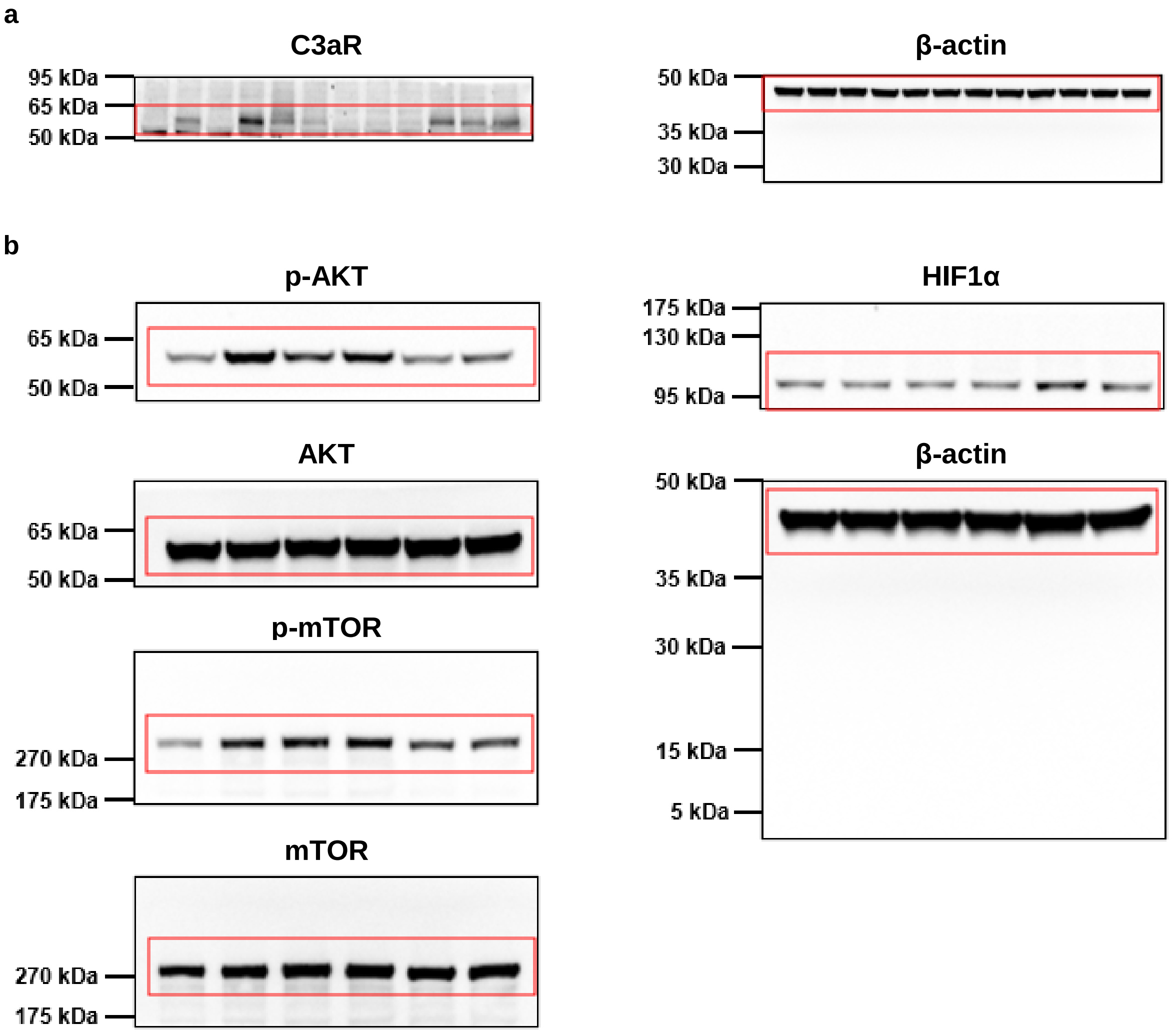

Additional File 3. Western Blot raw images. Blots used in Figure 4f (a) and Figure 5a (b) are shown. Red boxes indicate cropped areas shown in the figures.
