# Supplementary material for: Microglia undergo sex-dimorphic transcriptional and metabolic rewiring during aging
Source: J Neuroinflammation. 2024 Jun 5;21:150. doi: 10.1186/s12974-024-03130-7 (PMC11155174; doi:10.1186/s12974-024-03130-7)
Supplement: Supplementary file 4 — Supplementary Material 4 [file 12974_2024_3130_MOESM4_ESM.docx]

**Supplemental Figures**


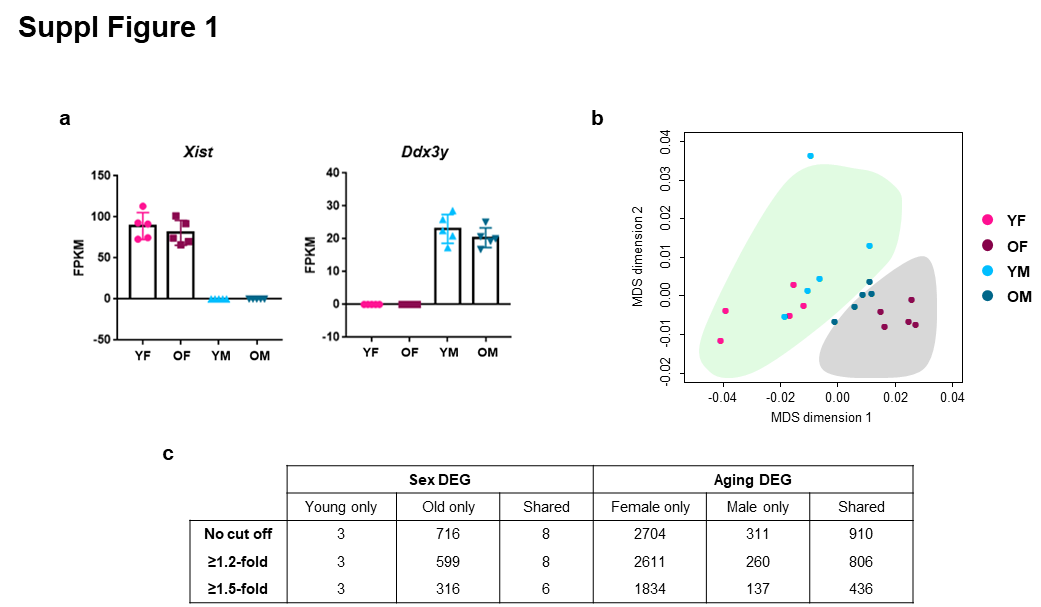


**Suppl. Figure 1. Transcriptomic profiling of hippocampal microglia in young and old, male and female mice.**

(a) Expression of *Xist* and *Ddx3y* verifies female and male sample identification, respectively. Data are presented as mean (SD) FPKM values. (b) Multidimensional scaling (MDS) analysis of bulk RNAseq data from young (4-month) and old (25-month), female and male microglia. (Y, young; O, old; F, female; M, male) (c) Pairwise comparisons showing number of differentially expression genes (DEG) calculated without fold change cut-offs and with 1.2- and 1.5-fold change cut-offs. Sex DEG = Female vs. Male; Aging DEG = Old vs. Young.


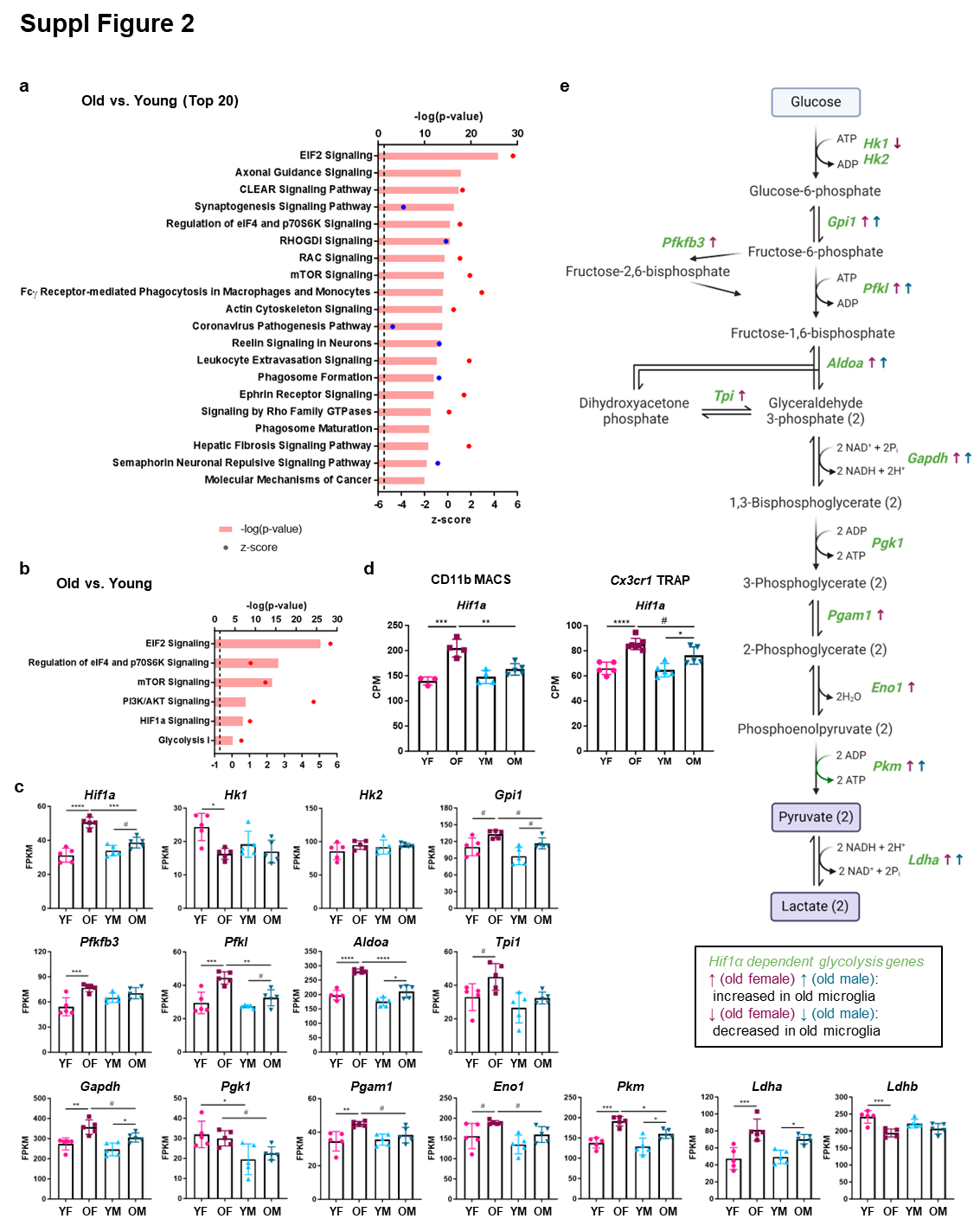


**Suppl. Figure 2. Expression of HIF1α-dependent glycolysis genes by hippocampal microglia.**

(a-b) Ingenuity Pathway Analysis (IPA) of aging-associated genes comparing old versus young (FDR < 5% with sex as a covariate). The top 20 most significant pathways (a) and metabolic pathways (b) are shown. Red dots represent positive z-scores, and blue dots represent negative z-scores. Dashed line: -log_10_ (p-value) cutoff of 1.3. (c) Expression *Hif1a* and glycolysis-related genes by young and old, female and male microglia. Data are presented as mean (SD) FPKM values. n=5/group. *p < 0.05, **p < 0.01, ***p < 0.001, ****p < 0.0001 (two-way ANOVA); #p < 0.05 (unpaired t-test). (d) *Hif1a* expression by hippocampal microglia from recently published datasets[21]: CD11b^+^ hippocampal microglia (RNAseq) and *Cx3cr1*-expressing hippocampal microglia (NuTRAP analysis of translating RNA). Data are presented as mean (SD) CPM values. n=3-5/group. *p < 0.05, **p < 0.01, ***p < 0.001, ****p < 0.0001 (two-way ANOVA); #p < 0.05 (unpaired t-test). (e) Schematic of the glycolysis pathway (created using BioRender.com). HIF1α-dependent glycolysis genes are shown in green. Dark pink arrows represent changes in old female microglia, and dark blue arrows represent changes in old male microglia.


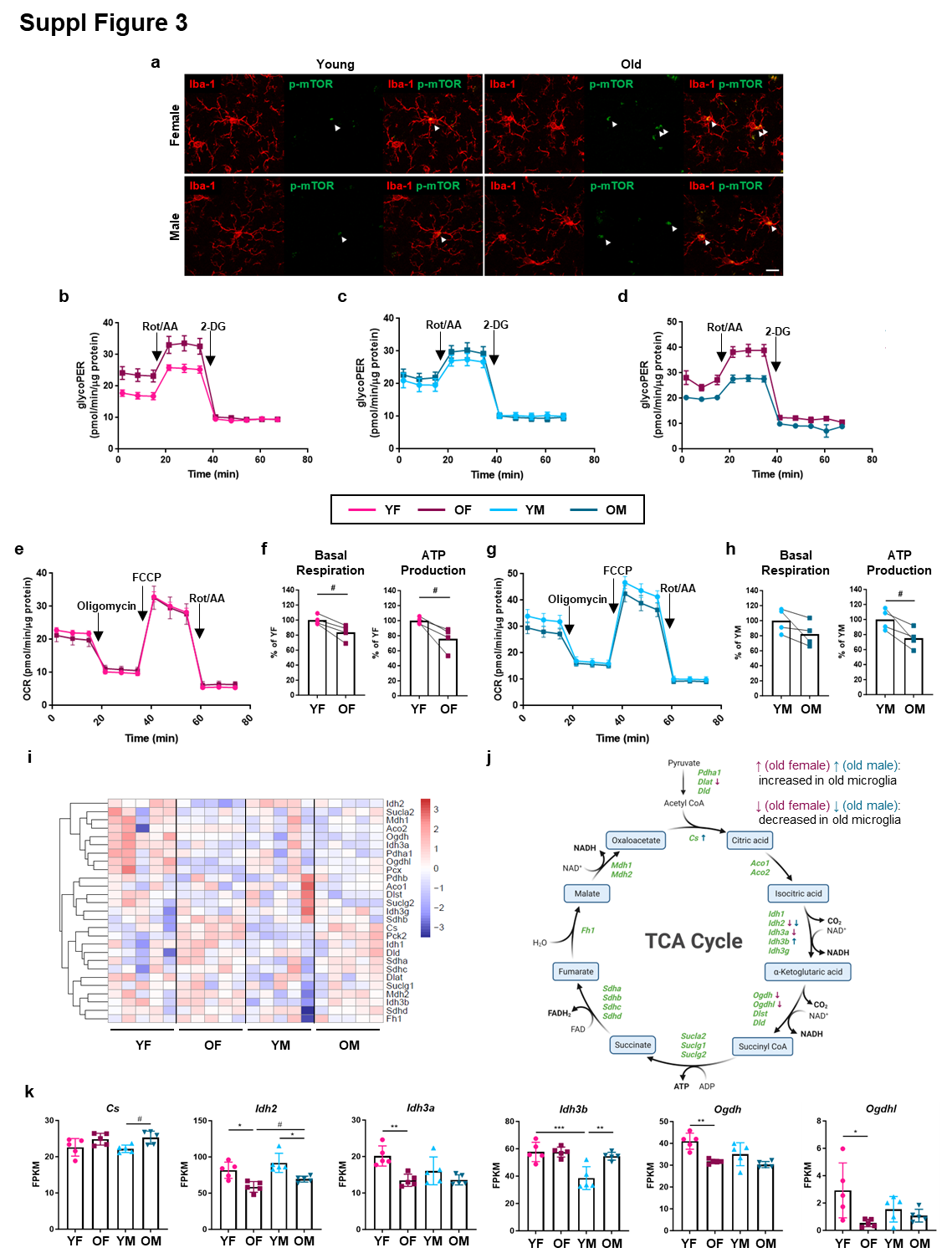


**Suppl. Figure 3. Metabolic analysis of cortical and hippocampal microglia.**

(a) Representative images (CA1 region) of mTOR phosphorylation in hippocampal microglia (Iba-1^+^). Scale bar = 10 μm. Arrow: co-localized Iba-1 and p-mTOR. (b-h) Microglia from the hippocampus and cortex were pooled and their metabolic activity was assessed using Seahorse assays. Basal and compensatory glycolysis were evaluated by treating with rotenone/antimycin A and 2-DG, and calculating the glycolytic Proton Efflux Rate (glycoPER) (b-d). Basal respiration and ATP production were evaluated by treating with oligomycin, FCCP, and rotenone/antimycin A, and calculating the oxygen consumption rate (OCR) (e-h). Stimuli were added as indicated in the figures. n=4/group. Old female microglia were compared to young female microglia (b, e, f), old male microglia were compared to young male microglia (c, g, h), and old female microglia were compared to old male microglia (d). Data are shown as mean (SEM) (b-e, g) or mean values of paired samples (f, h). #p < 0.05 (unpaired t-test). (i) Heatmap showing expression of genes in the TCA cycle KEGG pathway. Color bar indicates row z-score. (j) Schematic of the TCA cycle (created using BioRender.com). Genes involved at each step are shown in green. Dark pink arrows represent changes in old female microglia, and dark blue arrows represent changes in old male microglia. (k) Microglial expression of TCA cycle genes differentially expressed (FDR < 5%) by old female and/or old male microglia compared to their young counterparts. Data are presented as mean (SD) FPKM values. n=5/group. *p < 0.05, **p < 0.01, ***p < 0.001 (two-way ANOVA); #p < 0.05 (unpaired t-test).

**
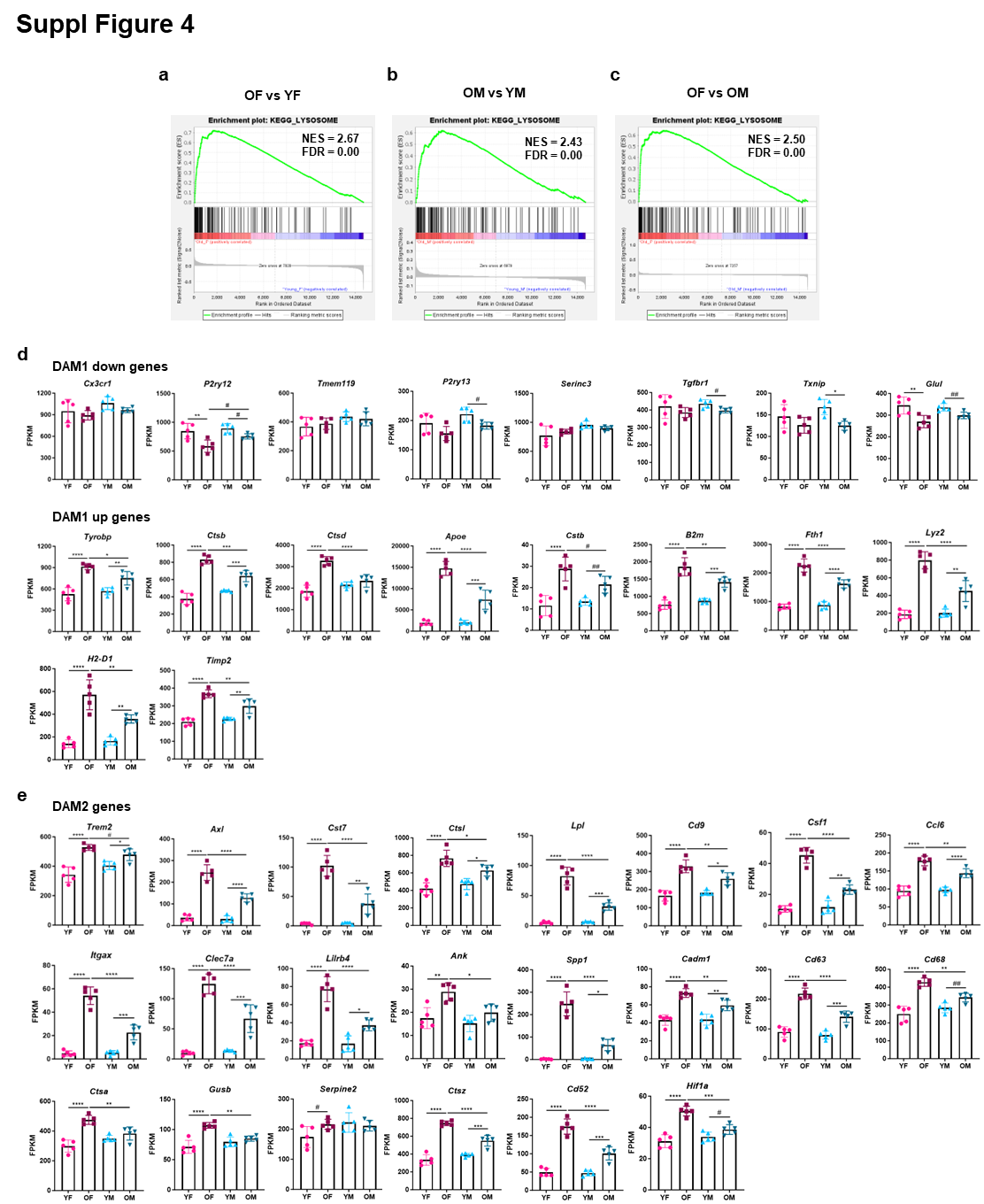
**

**Suppl. Figure 4. Expression of lysosomal pathway genes and DAM genes.**

(a-c) GSEA plots of the Lysosome KEGG pathway comparing old versus young female microglia (a), old versus young male microglia (b), and old female versus old male microglia (c). (d, e) Expression of DAM signature genes[7] by young and old, male and female hippocampal microglia assessed by bulk RNA-seq: genes downregulated and upregulated in DAM1 microglia compared to homeostatic microglia (DAM1 down and DAM1 up, respectively; d), and genes upregulated in DAM2 microglia compared to DAM1 microglia (e). Data are presented as mean (SD) FPKM values. n=5/group. *p < 0.05, **p < 0.01, ***p < 0.001, ****p < 0.0001 (two-way ANOVA); #p < 0.05, ##p < 0.01 (unpaired t-test). (note that *Cd68* data are also shown in Figure 3b, *Hif1a* data in Suppl. Figure 2c, and *Ctsl* data in Figure 4b)


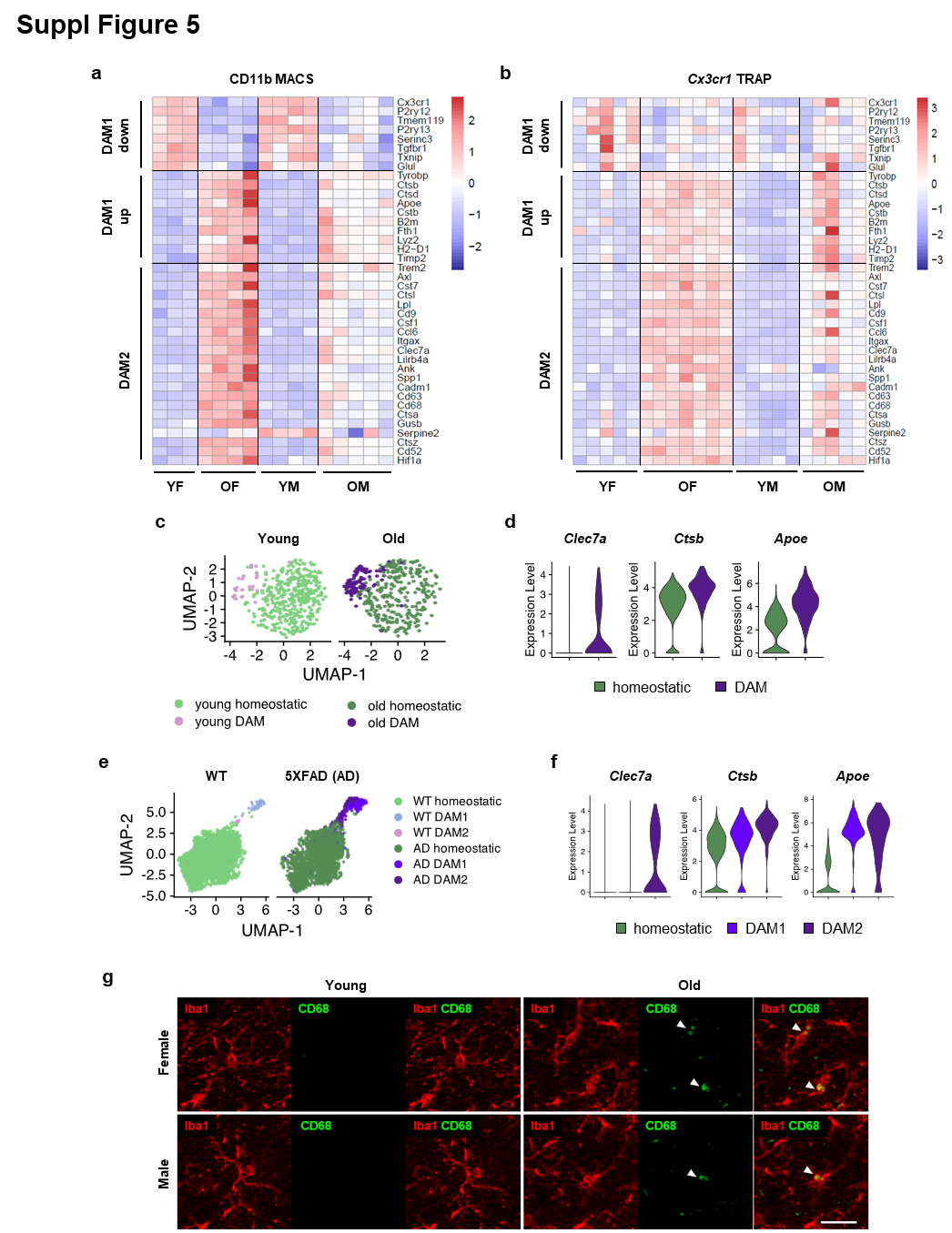


**Suppl. Figure 5. Analysis of homeostatic microglia and DAM in young and old mice and 5XFAD mice.**

(a, b) Heatmaps showing expression of genes downregulated and upregulated in DAM1 microglia compared to homeostatic microglia (DAM1 down and DAM1 up, respectively) in recently published datasets[21]: CD11b^+^ hippocampal microglia (RNAseq; a) and *Cx3cr1*-expressing hippocampal microglia (NuTRAP analysis of translating RNA; b). (c) UMAP of homeostatic microglia and DAM identified by scRNAseq (reanalyzed from [7]). (d) Expression of key DAM signature genes by the homeostatic microglia and DAM. (e) UMAP of homeostatic microglia and DAM (classified as DAM1 and DAM2) from WT and 5XFAD mice (reanalyzed from [7]). (f) Expression of key DAM signature genes by the homeostatic microglia and DAM1 and DAM2 cells. (g) Representative images of DAM (DG region) assessed by staining CD68 puncta in hippocampal microglia (Iba-1^+^). Scale bar = 20 μm. Arrow: co-localized Iba-1 and CD68.


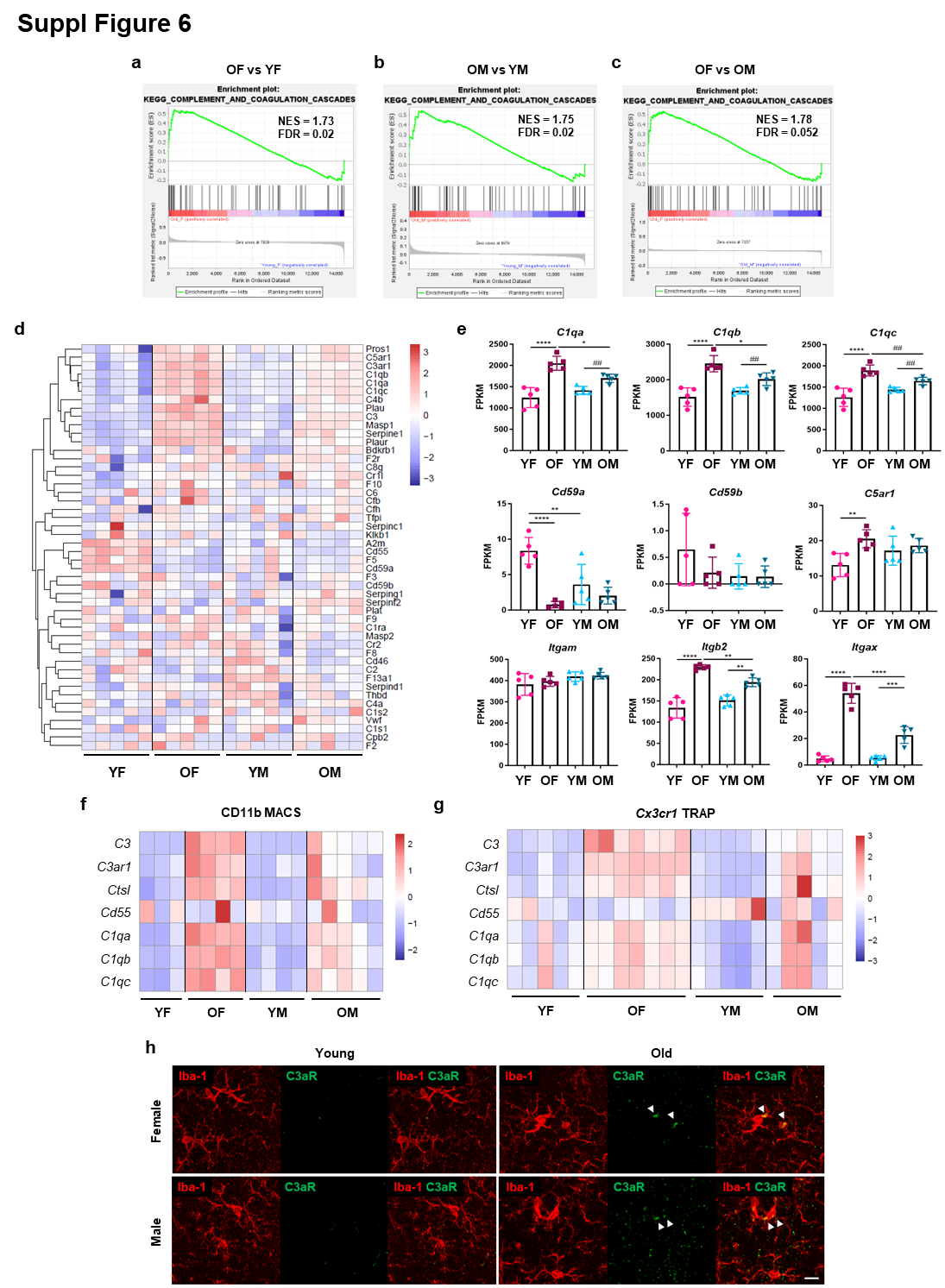


**Suppl. Figure 6. Expression of complement pathway genes.**

(a-c) GSEA plots of the KEGG Complement and Coagulation Cascades pathway comparing old versus young female microglia (a), old versus young male microglia (b), and old female microglia versus old male microglia (c) (FDR < 5%). NES, normalized enrichment score. (d) Heatmap of genes involved in the KEGG Complement and Coagulation Cascades pathway. Color bar indicates row z-score. (e) Expression of complement pathway-associated genes *C1qa*, *C1qb*, *C1qc*, *Cd59a*, *Cd59b*, *C5ar1*, *Itgam*, *Itgb2*, and *Itgax*. Data are presented as mean (SD) FPKM values. n=5/group. *p < 0.05, **p < 0.01, ***p < 0.001, ****p <0.0001 (two-way ANOVA); ##p < 0.01 (unpaired t-test). (f, g) Heatmaps of complement pathway gene expression by hippocampal microglia from recently published datasets[21]: CD11b^+^ hippocampal microglia (RNAseq; f) and *Cx3cr1*-expressing hippocampal microglia (NuTRAP analysis of translating RNA; g). (h) C3aR expression by microglia (Iba-1^+^) in the hippocampus. Representative images (CA1 region) are shown. Scale bar = 10 μm. Arrow: co-localized Iba-1 and C3aR.


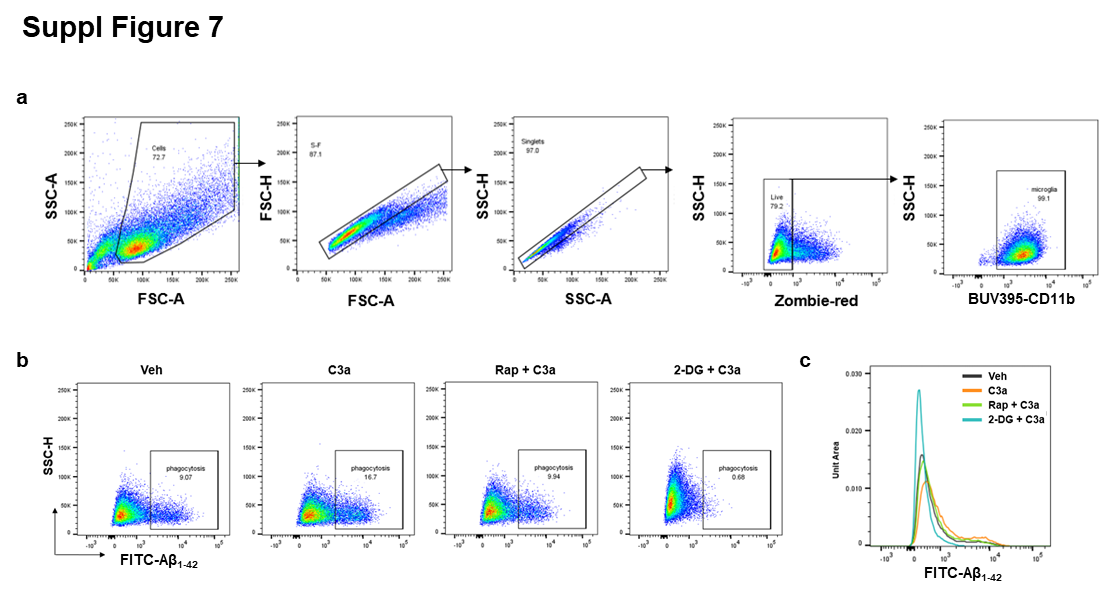


**Suppl. Figure 7. Evaluation of fAβ_1-42_ phagocytosis.**

Young microglia (postnatal day 0-2, pooled male and female) were treated with 10 nM recombinant mouse C3a for 18 h prior to addition of FITC-fAβ_1-42_ (1 μM). (a) Gating strategy of flow cytometry analysis using young microglia. CD11b^+^ live single cells (anti-CD11b BUV395) were analyzed for FITC-fAβ_1-42_ phagocytosis. (b) Representative dot plots showing the frequency of phagocytic microglia. Rapamycin (50 μM) or 2-DG (5 mM) were pre- (1 h) and co-treated (18 h) with C3a. (c) Representative histogram showing mean fluorescent intensity of FITC-fAβ_1-42_ inside the microglia.


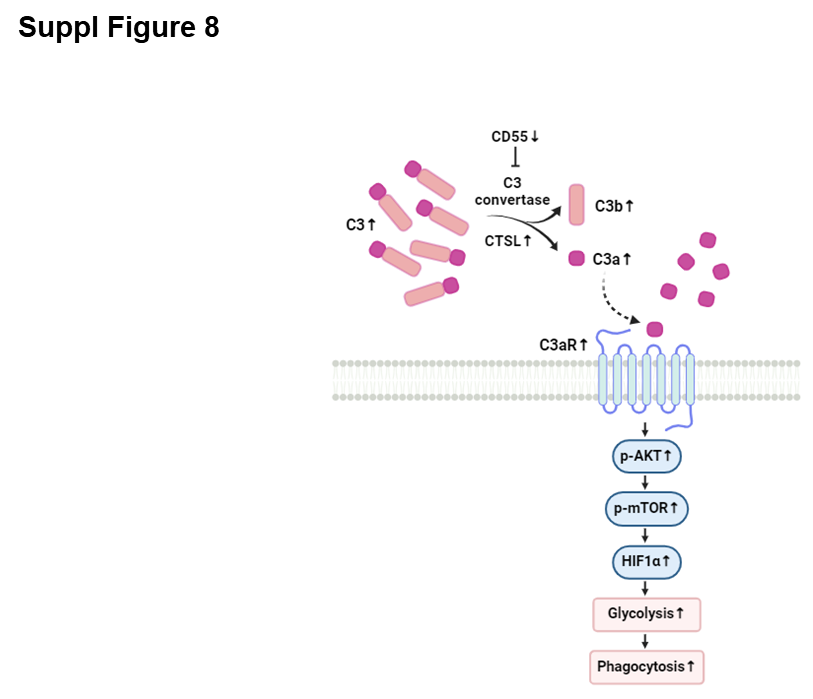


**Suppl. Figure 8. C3a-driven metabolic rewiring of DAM2 microglia.**

Our data support a model (created using BioRender.com) in which DAM2 microglia produce more C3a due to increased *C3* transcription and elevated cleavage of C3 to C3a and C3b. Moreover, increased C3aR expression results in stronger autocrine sensing of C3a, either at the cell surface or at an intracellular site, resulting in an AKT-mTOR-driven increase in HIF1α production that induces a metabolic shift to glycolysis and promotes their phagocytic activity.
